# Effectiveness of Aquatic Exercise and Balneotherapy: A Summary of Systematic Reviews Based on Randomized Controlled Trials of Water Immersion Therapies

**DOI:** 10.2188/jea.JE20090030

**Published:** 2010-01-05

**Authors:** Hiroharu Kamioka, Kiichiro Tsutani, Hiroyasu Okuizumi, Yoshiteru Mutoh, Miho Ohta, Shuichi Handa, Shinpei Okada, Jun Kitayuguchi, Masamitsu Kamada, Nobuyoshi Shiozawa, Takuya Honda

**Affiliations:** 1 Faculty of Regional Environment Science, Tokyo University of Agriculture, Tokyo, Japan; 2 Department of Drug Policy and Management, Graduate School of Pharmaceutical Sciences, The University of Tokyo, Tokyo, Japan; 3 Mimaki Onsen (Spa) Clinic, Tomi, Nagano, Japan; 4 Department of Physical and Health Education, Graduate School of Education, The University of Tokyo, Tokyo, Japan; 5 Laboratory of Aqua, Health, and Sports Medicine, Sapporo, Japan; 6 Physical Education and Medicine Research Foundation, Tomi, Nagano, Japan; 7 Physical Education and Medicine Research Center Unnan, Unnan, Shimane, Japan; 8 Department of Longevity and Social Medicine, Okayama University Graduate School of Medicine, Dentistry and Pharmaceutical Sciences, Okayama, Japan

**Keywords:** systematic review, aquatic exercise, randomized controlled trial, balneotherapy

## Abstract

**Background:**

The objective of this review was to summarize findings on aquatic exercise and balneotherapy and to assess the quality of systematic reviews based on randomized controlled trials.

**Methods:**

Studies were eligible if they were systematic reviews based on randomized clinical trials (with or without a meta-analysis) that included at least 1 treatment group that received aquatic exercise or balneotherapy. We searched the following databases: Cochrane Database Systematic Review, MEDLINE, CINAHL, Web of Science, JDream II, and Ichushi-Web for articles published from the year 1990 to August 17, 2008.

**Results:**

We found evidence that aquatic exercise had small but statistically significant effects on pain relief and related outcome measures of locomotor diseases (eg, arthritis, rheumatoid diseases, and low back pain). However, long-term effectiveness was unclear. Because evidence was lacking due to the poor methodological quality of balneotherapy studies, we were unable to make any conclusions on the effects of intervention. There were frequent flaws regarding the description of excluded RCTs and the assessment of publication bias in several trials. Two of the present authors independently assessed the quality of articles using the AMSTAR checklist.

**Conclusions:**

Aquatic exercise had a small but statistically significant short-term effect on locomotor diseases. However, the effectiveness of balneotherapy in curing disease or improving health remains unclear.

## INTRODUCTION

Aquatic exercise has been referred to as pool therapy, hydrotherapy, and, in earlier literature, sometimes even as balneotherapy.^1^ Exercise in warm water, usually called hydrotherapy or aquatic therapy, is a popular treatment for many patients with painful neurologic or musculoskeletal conditions.^2^ The warmth and buoyancy of water may block nociception by acting on thermal receptors and mechanoreceptors, thus influencing spinal segmental mechanisms.^3^^,^^4^ In addition, warm water may enhance blood flow, which is thought to help in dissipating algogenic chemicals, and facilitate muscle relaxation. In addition, the hydrostatic effect may relieve pain by reducing peripheral edema^5^ and by dampening sympathetic nervous system activity.^6^

Bathing in water (balneotherapy or spa therapy) without exercise has also been frequently used in alternative medicine as a disease cure. Spa therapy is a very popular form of treatment for all types of arthritis in many European countries, as well as in Israel and Japan.^7^^,^^8^ In addition, recent reports have demonstrated that comprehensive health education, which includes lifestyle education and exercise in combination with spa bathing, has positive effects for middle-aged and elderly people.^9^^,^^10^

Although many studies have reported the effects of water exercise and balneotherapy, there is no review of systematic reviews of evidence from randomized controlled trials. The objective of this review was to summarize evidence for the effectiveness of aquatic exercise and balneotherapy and to assess the quality of systematic reviews based on randomized controlled trials of these therapies.

## METHODS

### Criteria for study inclusion

#### Types of studies

Systematic reviews based on randomized clinical trials (with or without a meta-analysis) were eligible.

#### Types of participants

Studies were not excluded based on the disease status of participants (ill vs healthy people).

#### Types of intervention and language

Studies that included at least 1 treatment group in which aquatic exercise or balneotherapy were included. A study of any type of exercise used in a therapeutic indoor pool or bath (range of motion exercise, dynamic exercise, aerobic exercise, immersion only, etc.) was acceptable. Studies had to include information on use of medication, alternative therapies, and lifestyle changes, and these had to be comparable among groups. When comparing different programs, type of exercise, type of water, water depth, and water temperature were considered. There was no restriction on the basis of language.

### Methods used to identify studies

#### Bibliographic database

We searched the following databases: Cochrane Database Systematic Review, MEDLINE via PubMed from 1990, CINAHL from 1990, Web of Science from 1990, JDream II (in Japanese) from 1990, and Ichushi-Web (in Japanese) from 1990, for articles published up to August 17, 2008. The search was limited to studies published in or after 1990, the time period during which the systematic review methodology became accepted. All searches were performed by 2 hospital librarians who were qualified in medical information management and were highly trained in the retrieval of clinical trials.

#### Search strategies

The search strategies used for all databases contained the following elements and terms:

(I) Search “aquatic therapy” or “aquatic exercise” or “water exercise”
(II) Search (“water”[Majr] or “swimming”[Majr]) and exercise therapy/methods
(III) Search “water gymnastic” or “water training” or “water aerobics” or “pool exercise” or “pool therapy” or “aerobic aquatics” or “hydrotherapy” or “thalassotherapy” or “aquatics” or “balneotherapy” or “spa therapy”
(IV) Search I or II or III
(V) Search I or II or III Limits: systematic reviews/meta-analysis

Only keywords related to intervention were used for searching. First, titles and abstracts of identified published articles were reviewed to determine the relevance of the articles. Next, the references in relevant reviews and identified randomized controlled trials (RCTs) were screened.

#### Reference checking and hand searching

We did not check the references of included studies, nor did we perform any hand searches or contact institutions, societies, specialists with expertise in aquatic exercise or balneotherapy, or the authors of included studies to identify any additional published or unpublished data.

### Review methods

#### Selection of trials

**Figure 1.**
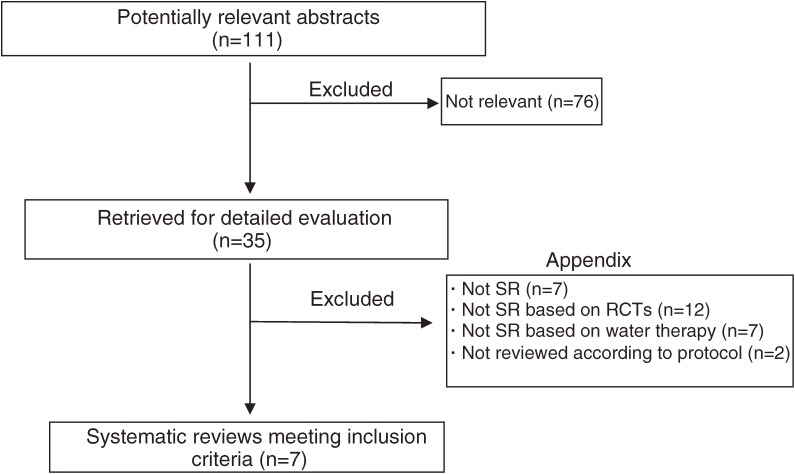
Flowchart of trial process SR: systematic review. RCT: randomized controlled trial.

For the final selection of studies for this review, 2 authors (HK and TH) independently applied all criteria to the full text of the articles that had passed the initial eligibility screening (Figure 1). Disagreements and uncertainties were resolved by discussion between the authors.

Studies were selected when (1) the design was a systematic review of RCTs, and (2) one of the interventions was a form of aquatic exercise or balneotherapy. Effectiveness of cure or health improvement was used as a primary outcome measure. Health improvement was defined broadly, and encompassed improvements in blood pressure, serum lipid profile, immunity, and quality of life. We excluded systematic reviews of non-RCTs or observational studies. Trials that were excluded are shown, along with the reason for exclusion, in the Appendix.

#### Quality assessment of included studies

To ensure that variation was not caused by systematic errors in study design or execution, 2 review authors (MK and HK) independently assessed the quality of articles. A full quality appraisal of these papers was made using the AMSTAR,^11^ which was developed to assess the methodological quality of systematic reviews. Disagreements and uncertainties were resolved by discussion between the review authors.

#### Summary of studies and data extraction

One author (HK) selected the summary from each of the structured abstracts and extracted the results for statistical analysis. The primary outcome measurement was always chosen for analysis.

#### Benefits and harms

The GRADE Working Group^12^ reported that the balance between benefits and harms, quality of evidence, applicability, and the probability of baseline risk were all considered in judgments of the strength of recommendations. Adverse events and withdrawals are particularly important for researchers and users of clinical practice guidelines, and we present this information with the description of each article.

## RESULTS

### Study characteristics

The literature searches identified 111 potentially relevant articles (Figure 1). Abstracts from those articles were assessed and 35 studies were retrieved for further evaluation (assessment of relevant literature). Twenty-eight publications were excluded either because they were not a systematic review (SR), not an SR based on RCTs, not an SR in which water was a factor, or were not reviewed according to protocol (see Appendix). Seven trials^1^^,^^2^^,^^13^^–^^17^ met all inclusion criteria (Tables 1 and 2). These included 3 SRs on aquatic exercise (spa therapy)^1^^,^^2^^,^^16^ and 5 SRs on balneotherapy^13^^–^^17^; one of these concerned both balneotherapy and spa therapy (with physiotherapy). The target diseases and disorders included knee and hip osteoarthritis,^1^^,^^14^^,^^15^ rheumatoid arthritis,^13^ low back pain,^16^ and neurologic or musculoskeletal disease (ie, rheumatoid arthritis, fibromyalgia, low back pain, and osteoarthritis), along with a number of other diseases and disorders.^2^ Studies on health improvement were also included.^17^ The SRs of aquatic exercise showed a curative effect in all studies; however, the SRs of balneotherapy provided no clear evidence of curative effect (Table 3).

**Table 1.** Summary of articles based on structured abstracts (aim and methods)

No.	Author	Journal Year; Vol.; Page.	Title	Aim/Objective	Data source/Search strategy	Selection criteria/period of intervention	Data extraction/Data collection and analysis
1	Bartels EM, et al.	Cochrane Database Syst Rev 2007;4:CD005523. (in English)	Aquatic exercise for the treatment of knee and hip osteoarthritis.	To compare the effectiveness and safety of aquatic exercise interventions in the treatment of knee and hip osteoarthritis.	MEDLINE from 1949, EMBASE from 1980, CENTRAL (Issue 2, 2006), CINAHL from 1982, Web of Science from 1945, all up to May 2006. There was no language restriction.	Randomized controlled trials or quasi-randomized clinical trials. The duration of interventions was from 6 weeks to 12 months.	Two review authors independently selected trials for inclusion, assessed the internal validity of included trials and extracted data. Pooled results were analyzed using standardized mean differences (SMD).

13	Verhagen AP, et al.	Cochrane Database Syst Rev 2008;3:CD000518. (in English)	Balneotherapy for rheumatoid arthritis.	To assess the effectiveness of balneotherapy for rheumatoid arthritis.	They searched the following databases up to October 2006: CENTRAL (Issue 3, 2006), PubMed, CINAHL, the database from the Cochrane “Rehabilitation and Related Therapies” Field and Pedro, and performed reference checking and personal communications with authors to retrieve eligible studies.	Selection criteria: randomized controlled trials comparing balneotherapy with any other intervention or with no intervention. Included patients were all suffering from definite or classical rheumatoid arthritis as defined by the American Rheumatism Association Criteria or by the criteria of Steinbrocker. At least one of the WHO/ILAR core set of endpoints for RA clinical trials had to be among the main outcome measures. The duration of interventions was from 14 weeks to 6 months and 4 weeks.	Two authors independently assessed quality and extracted data. Disagreements were solved by consensus.

14	Verhagen AP, et al.	Cochrane Database Syst Rev 2007;4:CD006864. (in English)	Balneotherapy for osteoarthritis.	To assess the effectiveness of balneotherapy for patients with osteoarthritis (OA).	They searched the following databases up to October 2006: EMBASE, PubMed, the Cochrane “Rehabilitation and Related Therapies” Field database, PEDro, CENTRAL (Issue 3, 2006), and performed reference checking and communicated with authors to retrieve eligible studies.	Randomized controlled trials (RCT) comparing balneotherapy with any intervention or no intervention. At least 90% of the patient population had to be diagnosed with osteoarthritis; duration of interventions was from 15 days to 27 weeks.	Two authors independently assessed quality and extracted data. Disagreements were solved by consensus. In the event of clinical heterogeneity or lack of data they refrained from statistical pooling.

15	Forestier R, et al.	Joint Bone Spine 2008;75:138–148. (in English)	Crenobalneotherapy for limb osteoarthritis: Systematic literature review and methodological analysis.	To conduct a systematic literature review on crenobalneotherapy for limb osteoarthritis and to discuss the study methods used to evaluate this treatment modality.	They searched the Medline database. They also reviewed the reference lists of articles retrieved by the Medline search. The studies had to be written in English or French.	Studies that compared crenobalneotherapy to other interventions or to no intervention were considered. Massage (usually an integral part of spa programs) is not specific to spa therapy and therefore was not studied here. Only studies of patients with osteoarthritis of the knee, hip, and/or hands were selected. The duration of interventions was from 16 days to 1 year and 3 months.	They used a checklist specifically designed to evaluate the internal validity of nonpharmacological trials. External validity and the quality of the statistical analysis were also evaluated.

16	Pittler MH, et al.	Rheumatol 2006;45:880–883. (in English)	Spa therapy and balneotherapy for treating low back pain: meta-analysis of randomized trials.	To assess the evidence for or against the effectiveness of spa therapy and balneotherapy for treating low back pain.	Systematic searches were conducted on Medline, Embase, Amed Cochrane Central, the UK National Research Register and ClincalTrials.gov (all until July 2005).	All trials reporting that the sequence of allocation was randomized (RCTs). Testing balneotherapy or spa therapy for treating patients with low back pain were included. Trials reported in duplicate were excluded. The duration of interventions was from 3 weeks to 4 weeks.	Data abstraction was performed systematically and independently according to design, quality, sample size, intervention, water characteristics, results, adverse events and concomitant treatment.

2	Hall J, et al.	Arch Phys Med Rehabil 2008;89:873–883. (in English)	Does aquatic exercise relieve pain in adults with neurologic or musculoskeletal disease? A systematic review and meta-analysis of randomized controlled trials.	To evaluate the literature on the effectiveness of aquatic exercise in relieving pain in adults with neurologic or musculoskeletal disease.	A systematic literature search of 14 databases was examined for research on aquatic exercise over the period from January 1980 to June 2006.	Randomized controlled trials (RCTs) that included adults with neurologic or musculoskeletal disease, pain as an outcome measure, and exercise in water were included. The duration of interventions was from 4 weeks to 12 months.	Information on the participants, interventions, and outcomes was extracted from the included studies. Quality appraisal was assessed using the Scottish Intercollegiate Guidelines Network criteria for RCTs.

17	Kamioka H, et al.	J Jpn Soc Balneol Climatol Phys Med 2006;69:155–166. (in Japanese with English abstract)	A systematic review of randomized controlled trials on the therapeutic and health-promoting effects of spas.	To review randomized controlled trials of the effects of treatment in spas, thereby clarifying therapeutic effects of these treatments on individual diseases, and its health-promoting effects.	They searched the PubMed database twice: in Sept. 2004 and in April 2005. Articles published after 1990 and written in English were searched.	Key words for study selection were “randomized controlled trial” and “spa” or “balneotherapy”. No criteria were set up concerning the number of subjects, the observation period, or the kind of disease studied. The duration of interventions was from 3 weeks to 12 months.	The quality of individual articles was evaluated on a 13-point modified PEDro scale that was constructed by adding three terms, representing the number of subjects, the observation period, and water characteristics to the 10-point PEDro scale.

**Table 2.** Summary of articles based on structured abstracts (results and conclusion)

No.	Author	Main results	Conclusion
1	Bartels EM, et al.	In total, six trials (800 participants) were included. At the end of treatment for combined knee and hip osteoarthritis, there was a small-to-moderate effect on function (SMD 0.26, 95% confidence interval (CI) 0.11 to 0.42) and a small-to-moderate effect on quality of life (SMD 0.32, 95% CI 0.03 to 0.61). A minor effect of a 3% absolute reduction (0.6 fewer points on a 0 to 20 scale) and 6.6% relative reduction from baseline was found for pain. Only two studies reported adverse effects, that is, the interventions did not increase self-reported pain or symptom scores.	Aquatic exercise appears to have some beneficial short-term effects for patients with hip and/or knee OA; no long-term effects were documented. The controlled and randomized studies in this area are still too few to give further recommendations on how to apply the therapy, and studies of clearly defined patient groups with long-term outcomes are needed.

13	Verhagen AP, et al.	One extra study is included in this update. Now seven trials (412 patients) were included in this review. Most trials reported positive findings on their main outcomes, but were methodologically flawed to some extent. A ‘quality of life’ outcome was reported by two trials. None of the trials performed an intention-to-treat analysis and only two performed a comparison of effects between groups. Pooling of the data was not performed because of heterogeneity of the studies, multiple outcome measurements, and the overall poor data presentation. We found a significant benefit of mineral baths compared to Cyclosporine A at eight weeks on pain in one study (RR = 2.4; 95% CI: 1.4, 3.8). Overall there is insufficient evidence that balneotherapy is more effective than no treatment, that one type of bath is more effective than another, or that one type of bath is more effective than mudpacks, exercise, or relaxation therapy.	Silver level evidence was found for one study in favor of mineral baths compared to drug treatment at eight weeks. Insufficient evidence was found for all other comparisons. However the scientific evidence is insufficient because of poor methodological quality. Therefore, the noted “positive findings” should be viewed with caution.

14	Verhagen AP, et al.	Seven trials (498 patients) were included in this review. Two studies compared spa treatment with no treatment. One study evaluated baths as an add-on treatment to home exercise and the author compared thermal water from Cserkeszolo with tap water (placebo). Three studies compared sulphur or Dead Sea baths with no treatment or mineral baths with tap water baths or no treatment. Only one of the trials performed an intention-to-treat analysis and two studies provided enough data to perform our own intention-to-treat analysis. A ‘quality of life’ outcome was reported by one trial.	They found silver level evidence concerning the beneficial effects of mineral baths compared to no treatment. Regarding all other balneological treatments, no clear effects were found. However, the scientific evidence is weak because of the poor methodological quality and the absence of adequate statistical analysis and data presentation. The noted “positive findings” should be viewed with caution.

15	Forestier R, et al.	Crenobalneotherapy was associated with improvements in the evaluation criteria (pain, function, and quality of life) compared to baseline. However, inadequate internal validity precluded the establishment of a causal link between these improvements and crenobalneotherapy. External validity was often poorly defined. Some studies found no significant differences with the control group but failed to include a sample-size calculation, suggesting inadequate statistical power as a possible explanation for the result. In several studies, the use of multiple evaluation criteria and measurements led to a high risk of Type I error.	Although the consistency of the results suggests a therapeutic effect of crenobalneotherapy in limb osteoarthritis, available studies are methodologically inadequate and sample sizes too small to allow definitive conclusions. They suggest a number of solutions to these shortcomings. Carefully designed studies in larger patient populations are needed to determine the role of crenobalneotherapy in knee osteoarthritis.

16	Pittler MH, et al.	Five randomized clinical trials met all inclusion criteria. Quantitative data synthesis was performed. The data for spa therapy, assessed on a 100-mm visual analogue scale (VAS), suggest significant beneficial effects compared with waiting list control groups (weighted mean difference 26.6 mm, 95% confidence interval 20.4–32.8, *n* = 442) for patients with chronic low back pain. For balneotherapy, the data, assessed on a 100-mm VAS, also suggest beneficial effects compared with control groups (weighted mean difference 18.8 mm, 95% confidence interval 10.3–27.3, *n* = 138).	Even though the data are scarce, there is encouraging evidence suggesting that spa therapy and balneotherapy may be effective for treating patients with low back pain. These data are not compelling but warrant rigorous large-scale trials.

2	Hall J, et al.	Nineteen studies met the inclusion criteria; 8 had a moderate-to-low risk of bias, and 5 of these had data suitable for meta-analyses. This showed that aquatic exercise has a small posttreatment effect in relieving pain compared with no treatment (*P* = .04; standardized mean difference [SMD], −.17; 95% confidence interval [CI], −.33 to −.01), but it is not possible to draw a firm conclusion because of the lack of consistency of evidence across studies. Comparable pain-relieving effects were found between aquatic and land-based exercise (*P* = .56; SMD = .11; 95% CI, −.27 to .50).	There is sound evidence that there are no differences in pain-relieving effects between aquatic and land exercise. Compared with no treatment, aquatic exercise has a small pain-relieving effect; however, the small number of good-quality studies and inconsistency of results means that insufficient evidence limits firm conclusions.

17	Kamioka H, et al.	A total of 17 articles were reviewed. Diseases studied in these articles were mostly locomotor disorders, with pain as a main symptom: rheumatism, osteoarthritis, lumbago, Parkinson’s disease, varicosis, psoriasis, and health-promotion. The mean score on the 13-point modified PEDro scale was 7.5 (SD, 2.3), with a minimum score of 2 points and a maximum score of 12 points. In addition to balneotherapy, exercise therapy, mud pack treatment, and douche massage were employed in numerous studies. Improvements in the indicators were always more marked in balneotherapy intervention groups than in control groups, irrespective of the disease studied.	They devised a “3-layer model of evidence to be accumulated in balneotherapy” and concluded that RCT quality, evidence level, and expectation of good results were high for, in descending order, pain-relieving effect, functional recovery and improvement in quality of life, and health-promoting effects.

**Table 3.** Brief summary of 7 systematic reviews

No.	Author	Year of publication	Intervention type	Meta-analysis	Object disease	Effects noted
1	Bartels EM, et al.	2007	Aquatic exercise	Performed	Hip and knee Osteoarthritis	Short-term effects

13	Verhagen AP, et al.	2008	Balneotherapy	Not performed	Rheumatoid arthritis	Unclear, but effects in some trials

14	Verhagen AP, et al.	2007	Balneotherapy	Not performed	Osteoarthritis	Unclear, but effects in some trials

15	Forestier R, et al.	2008	Balneotherapy	Not performed	Limb osteoarthritis	Unclear, but effects in some trials

16	Pittler MH, et al.	2006	Balneotherapy and aquatic exercise	Performed	Low back pain	Effect for both interventions

2	Hall J, et al.	2008	Aquatic exercise	Performed	Neurologic or musculoskeletal disease	Small effect

17	Kamioka H, et al.	2006	Balneotherapy	Not performed	Locomotor disease and health improvement	Unclear, but effects in some trials

### Results of meta-analysis

Only 3 SRs^1^^,^^2^^,^^16^ provided data that were suitable for statistical pooling. Regarding the effectiveness of aquatic exercise for the treatment of knee and hip osteoarthritis,^1^ there was a small but statistically significant favorable effect for aquatic exercise on function (*P* < 0.001; weighted standardized mean difference [SMD], 0.26; 95% confidence interval [CI], 0.11 to 0.42; *n* = 648), quality of life (*P* < 0.05; SMD, 0.32; 95% CI, 0.03 to 0.61; *n* = 599), and mental health (*P* < 0.05; SMD, 0.16; 95% CI, 0.01 to 0.32; *n* = 642) measured immediately after the intervention period. Pain was assessed using a 100-mm visual analogue scale (VAS). A 3% absolute reduction and 6.6% relative reduction from baseline were found for pain (*P* < 0.05; SMD, 0.19; 95% CI, 0.04 to 0.35; *n* = 638). No statistically significant differences were found for walking ability or stiffness.

Next, we examined the effectiveness of aquatic exercise for pain relief.^2^ Aquatic exercise was significantly inversely associated with pain (*P* < 0.05; SMD, −0.17; 95% CI, −0.33 to −0.01; *n* = 594). However, meta-analysis showed no differences between aquatic exercise and land exercise (*P* = 0.56; SMD, 0.11; 95% CI, −0.27 to 0.50; *n* = 103).

We then examined the effectiveness of spa therapy (with physiotherapy) and balneotherapy for treating low back pain.^16^ Pain was assessed using a 100-mm VAS. Spa therapy was significantly inversely associated with pain (*P* < 0.001; SMD, 26.6; 95% CI, 20.4 to 32.8; *n* = 442), as was balneotherapy (*P* < 0.001; SMD, 18.8; 95% CI, 10.3 to 27.3; *n* = 138). Results on the Schober index and assessment of lumbar flexibility suggested there were no significant intergroup differences.

### Withdrawals and adverse events

Withdrawals (dropouts) were reported in 3 studies, and adverse events were reported in 4 studies (Table 4). No fatal accidents or serious adverse effects were noted in studies that reported adverse events.

**Table 4.** Description of adverse events and withdrawals in articles

No.	Author	Title	Withdrawals (dropouts) described?	Adverse events described?
1	Bartels EM, et al.	Aquatic exercise for the treatment of knee and hip osteoarthritis	Yes	Yes

14	Verhagen AP, et al.	Balneotherapy for rheumatoid arthritis	Yes	Yes

15	Verhagen AP, et al.	Balneotherapy for osteoarthritis	Yes	Yes

16	Forestier R, et al.	Crenobalneotherapy for limb osteoarthritis: Systematic literature review and methodological analysis	Yes	No

17	Pittler MH, et al.	Spa therapy and balneotherapy for treating low back pain: meta-analysis of randomized trials	No	Yes

2	Hall J, et al.	Does aquatic exercise relieve pain in adults with neurologic or musculoskeletal disease? A systematic review and meta-analysis of randomized controlled trials	Yes	Yes

18	Kamioka H, et al.	A systematic review of randomized controlled trials on the therapeutic and health-promoting effects of spas	No	No

### Quality assessment

A list of excluded studies (3 trials, 43%) and the use of graphic aids to assess publication bias (1 trial, 14%) were evaluated by using the AMSTAR checklist (Table 5).

**Table 5.** Evaluation of the quality of systematic reviews by using the AMSTAR checklist^11^

No.	Items	Answer	*n*	(%)
1.	Was an ‘a priori’ design provided? The research question and inclusion criteria should be established before the conduct of the review.	Yes	7	(100)
		No	0	(0)
		Can’t answer	0	(0)
		Not applicable	0	(0)

2.	Was there duplicate study selection and data extraction? There should be at least two independent data extractors and a consensus procedure for disagreements should be in place.	Yes	6	(86)
		No	0	(0)
		Can’t answer	1	(14)
		Not applicable	0	(0)

3.	Was a comprehensive literature search performed? At least two electronic sources should be searched. The report must include years and databases used (e.g., Central, EMBASE, and MEDLINE). Key words and/or MESH terms must be stated and where feasible the search strategy should be provided. All searches should be supplemented by consulting current contents, reviews, textbooks, specialized registers, or experts in the particular field of study, and by reviewing the references in the studies found.	Yes	5	(71)
		No	2	(29)
		Can’t answer	0	(0)
		Not applicable	0	(0)

4.	Was the status of publication (i.e., grey literature) used as an inclusion criterion? The authors should state that they searched for reports regardless of their publication type. The authors should state whether or not they excluded any reports (from the systematic review), based on their publication status, language etc.	Yes	7	(100)
		No	0	(0)
		Can’t answer	0	(0)
		Not applicable	0	(0)

5.	Was a list of studies (included and excluded) provided? A list of included and excluded studies should be provided.	Yes	3	(43)
		No	4	(57)
		Can’t answer	0	(0)
		Not applicable	0	(0)

6.	Were the characteristics of the included studies provided? In an aggregated form such as a table, data from the original studies should be provided on the participants, interventions and outcomes. The ranges of characteristics in all the studies analyzed e.g., age, race, sex, relevant socioeconomic data, disease status, duration, severity, or other diseases should be reported.	Yes	7	(100)
		No	0	(0)
		Can’t answer	0	(0)
		Not applicable	0	(0)

7.	Was the scientific quality of the included studies assessed and documented? ‘A priori’ methods of assessment should be provided (e.g., for effectiveness studies if the author(s) chose to include only randomized, double-blind, placebo controlled studies, or allocation concealment as inclusion criteria); for other types of studies alternative items will be relevant.	Yes	7	(100)
		No	0	(0)
		Can’t answer	0	(0)
		Not applicable	0	(0)

8.	Was the scientific quality of the included studies used appropriately in formulating conclusions? The results of the methodological rigor and scientific quality should be considered in the analysis and the conclusions of the review, and explicitly stated in formulating recommendations.	Yes	7	(100)
		No	0	(0)
		Can’t answer	0	(0)
		Not applicable	0	(0)

9.	Were the methods used to combine the findings of studies appropriate? For the pooled results, a test should be done to ensure the studies were combinable, to assess their homogeneity (i.e. Chi-squared test for homogeneity). If heterogeneity exists a random effects model should be used and/or the clinical appropriateness of combining should be taken into consideration (i.e., is it sensible to combine?).	Yes	5	(71)
		No	1	(14)
		Can’t answer	1	(14)
		Not applicable	0	(0)

10.	Was the likelihood of publication bias assessed? An assessment of publication bias should include a combination of graphical aids (e.g., funnel plot, other available tests) and/or statistical tests (e.g., Egger regression test).	Yes	1	(14)
		No	6	(86)
		Can’t answer	0	(0)
		Not applicable	0	(0)

11.	Was the conflict of interest stated? Potential sources of support should be clearly acknowledged in both the systematic review and the included studies.	Yes	5	(71)
		No	2	(29)
		Can’t answer	0	(0)
		Not applicable	0	(0)

## DISCUSSION

We identified only 7 published SRs on aquatic exercise and balneotherapy, which indicates that there is little evidence demonstrating the effectiveness of the warmth, buoyancy, and hydrostatic effects of water for curing disease or improving health. One reason for the limited number of SRs may be that aquatic exercise and balneotherapy are similar practices and distinguishing between them in RCTs is thus difficult. In addition, participants may find the intervention process, which requires them to undress and wear a swimsuit, to be troublesome. Furthermore, it is difficult to perform meta-analyses because, in the case of balneotherapy, the chemical content and temperature of the waters studied differ in various countries and the data are therefore not easily integrated.

### Aquatic exercise versus balneotherapy (without exercise)

We distinguished between aquatic exercise and balneotherapy to determine which was more effective, because many studies do not do so. Aquatic exercise had a small but statistically significant effect on pain, function, QOL and mental health, and included more voluntary movements during water immersion. This suggests that an intervention requiring exercise is more effective for the treatment of musculoskeletal diseases, as compared to balneotherapy, which involves passive immersion. However, it should be noted that this was only the immediate effect of intervention, and not the long-term result. The intervention period ranged from 3 weeks to 12 months in aquatic exercise studies, and from 15 days to 12 months in studies of balneotherapy. This might reflect the difficulty of maintaining long-term participation in an RCT. Whatever the case, the long-term effects are not clear.

We did not pool data from SRs of balneotherapy^13^^–^^15^^,^^17^ because of their heterogeneity, multiple and varied outcome measurements, and poor overall quality. SRs of balneotherapy suggested that the scientific evidence was insufficient because of the poor methodological quality of RCTs of balneotherapy. Thus, it is difficult to determine the independent effect of balneotherapy without exercise.

### Quality assessment

Seven of the included SRs were published after 2006 and, hence, relatively recent. We used the AMSTAR checklist because its content validity is high and the number of articles reviewed to evaluate SR quality was as few as 11. The requirements of the AMSTAR checklist were generally satisfied; however, an assessment of publication bias was frequently omitted. The AMSTAR requires that an assessment of publication bias include a combination of graphic aids (eg, funnel plot other available tests). One important publication reported that authors were more likely to publish RCTs in an English-language journal if the results were statistically significant.^18^ English language bias may therefore be present in reviews and meta-analyses that include only trials reported in English.

There were few lists of excluded studies: only 3 Cochrane Reviews^1^^,^^13^^,^^14^ reported this information. If the format of SRs adheres to that of the Cochrane Review, recording omissions would be minimal. However, many scientific journals limit the length of submissions, so such descriptions may not be published. We believe it is necessary to include a list of excluded studies in order to improve the certainty and transparency of studies.

### Overall evidence and future research agenda

Table 6 shows the overall evidence and future research agenda for aquatic exercise and balneotherapy. Aquatic exercise had a small but statistically significant effect. Future RCTs should investigate the long-term effectiveness of aquatic exercise or its effectiveness with respect to type or duration of exercise. Then, SRs based on such RCTs can be conducted. Regarding balneotherapy, RCTs based on appropriate research methodology are needed because no clear effect was found in the present study. A common problem with RCTs is that they do not properly evaluate adverse effects; future studies should include these data.

**Table 6.** Overall evidence and future research agenda

Intervention	Evidence	Specific agenda	Common agenda
Aquatic exercise	Small but significant effect (no differences between aquatic exercise and land exercise)	1. Long-term effect 2. Type of dose (intensity, frequency and duration)	1. Randomized controlled trials for various diseases 2. Cost-benefit analysis 3. Description of adverse effects

Balneotherapy	Poor/Unclear	Satisfactory methodology (intention-to-treat analysis, blinding, adequate control group, etc.)	

A recent study suggested that the most important questions that authors of systematic reviews face are as follows^19^: (1) How can incorporating existing reviews into new work adhere to the principles of comprehensive, transparent, and unbiased methods required for systematic reviews? (2) If an effort is made to incorporate existing reviews, will it save time and resources? (3) Are there instances where an independent, critical assessment of the evidence warrants conducting a complex review “from scratch” even if there are existing reviews?

### Study limitations

There were several limitations to the present study. Some selection criteria were common to the studies, as described above; however, bias remained due to differences in the eligibility for participation in each study.

Publication bias was also a limitation. Although we did not limit our search to English language articles, we found no articles published in other languages. Also, we were not able to check references by means of hand searches. Nor were we able to contact institutions, societies, or specialists with expertise in aquatic exercise or balneotherapy or authors of included studies to identify any additional published or unpublished data. Another limit of the study was that we were not able to search the PEDro database, which is used in fields such as rehabilitation medicine and physiotherapy.

In terms of quality assessment, disagreements and uncertainties were resolved by discussion between 2 authors; discussions with a third expert and contact with authors for the purpose of clarification were not allowed.

### Conclusion

There were relatively few SRs of RCTs on aquatic exercise and balneotherapy. We found that aquatic exercise had a small but statistically significant effect on pain relief and related outcome measurements for locomotor diseases. However, the long-term effectiveness of these treatments remains unclear.

Because there was insufficient evidence due to the poor methodological quality of balneotherapy studies, we are unable offer any conclusions about the effects of this intervention. Common flaws included an inadequate description of excluded RCTs and insufficient assessment of publication bias.

**Appendix.** Studies excluded in the present review

No.	Author. Journal (Year)	Title	Reason for exclusion
E1	Cardoso JR, et al. Cochrane Database Systc Rev (2008)	Aquatic therapy exercise for treating rheumatoid arthritis (Protocol)	Not reviewed due to protocol

E2	Beamon S, et al. Cochrane Database Syst Rev (2008)	Hydrotherapy for asthma (Protocol)	Not reviewed due to protocol

E3	Dziedzic K, et al. Best Practice Research Clin Rheumatol (2008)	Land- and water-based exercise therapies for musculoskeletal condition	Not SR

E4	Getz M, et al. Clin Rehabili (2006)	Effects of aquatic interventions in children with neuromotor impairments: a systematic review of the literature	Not SR based on RCTs

E5	Tejirian T, et al. Diseases Colon Rectum (2005)	Sitz bath: where is the evidence? Scientific basis of a common practice	Not SR

E6	Herman PM, et al. BMC Complementary Alternative Med (2005)	Is complementary and alternative medicine (CAM) cost-effective? a systematic review	Not SR based on water

E7	Karagulle MZ, et al. Forsch Komplementarmed Klass Naturheilkd (2004)	Balneotherapy and spa therapy of rheumatic diseases in Turkey: a systematic review (in German)	Not SR based on RCTs

E8	Meremikwu M, et al. Cochrane Database Syst Rev (2008)	Physical methods for treating fever in children	Not SR based on water

E9	Liao WC. Int J Nursing Studies (2002)	Effects of passive body heating on body temperature and sleep regulation in the elderly: a systematic review	Not SR based on RCTs

E10	Pennick VE, et al. Cochrane Database Syst Rev (2007)	Interventions for preventing and treating pelvic and back pain in pregnancy	Not SR based on water

E11	Teschendorf ME, et al. Am J Maternal/Child Nursing (2000)	Hydrotherapy during labor: an example of developing a practice policy	Not SR based on RCTs

E12	Verhagen AP, et al. J Rheumatol (1997)	Taking baths: the efficacy of balneotherapy in patients with arthritis. A systematic review	Not SR based on RCTs

E13	Sim J, et al. Clin J Pain (2002)	Systematic review of randomized controlled trials of nonpharmacological interventions for fibromyalgia	Not SR based on water

E14	Rosimini C, et al. J Am Academy Nurse Practitioners (2003)	Benefits of swim training for children and adolescents with asthma	Not SR based on RCTs

E15	Schiltenwolf M, et al. Schmerz (2008)	Physiotherapy, exercise and strength training and physical therapies in the treatment of fibromyalgia syndrome (in German)	Not SR based on RCTs

E16	Toumaire M, et al. e CAM (2007)	Complementary and alternative approaches to pain relief during labor	Not SR

E17	Bouchama A, et al. Critical Care (2007)	Cooling and hemodynamic management in heatstroke: practical recommendations	Not SR based on RCTs

E18	Iarustovskaia OV, et al. Vopr Kurotol Fizioter Lech Fiz Kult (2006)	Thermocontrast hydrotherapy in the treatment of neuroendocrine disorders in females of reproductive age (in Russian)	Not SR based on RCTs

E19	Balint G, et al. Orv Hetil (2006)	Rehabilitation and balneotherapy, wellness 2004 (in Hungarian)	Not SR based on RCTs

E20	Adilov VB, et al. Vopr Kurotol Fizioter Lech Fiz Kult (2006)	Mineral waters for external (balneological) application. Guide for physicians (in Russian)	Not SR

E21	Markel W. Wien Kiln Wochenschr (2006)	Can the effects of radon therapy be scientifically substantiated? (in Russian)	Not SR

E22	Getenbrunner C. Wien Klin Wochenschr (2006)	Could balneology and medical climatology have more than historic importance in the therapy of chronic diseases? (in Russian)	Not SR

E23	Davydova DB, et al. Vopr Kurotol Fizioter Lech Fiz Kult (2006)	Hydrobalneotherapy of patients with cardiovascular disease. Manual for physicians (in Russian)	Not SR

E24	Hodgson S. Clin Orthopaedics Related Research (2006)	Proximal humerus fracture rehabilitation	Not SR based on RCTs

E25	Liu Y, et al. Current Opinion Rheumatol (2004)	Recent advances in the treatment of the spondyloarthropathies	Not SR based on water

E26	Watts R, et al. Int J Nursing Practice (2003)	Nursing management of fever in children: a systematic review	Not SR based on water

E27	Pengel HM, et al. Clin Rehabili (2002)	Systematic review of conservative interventions for subacute low back pain	Not SR based on water

E28	Constant F, et al. Bull Soc Sci Med Grand Duche Luxemb (1995)	Critical bibliographic analysis of international medical literature in the domain of thermal research	Not SR based on RCTs
